# Comparison of Outcomes Between Angiotensin-Converting Enzyme Inhibitors and Angiotensin II Receptor Blockers in Patients With Myocardial Infarction: A Meta-Analysis

**DOI:** 10.7759/cureus.47954

**Published:** 2023-10-30

**Authors:** Johao Escobar, Anurag Rawat, Fabricio Maradiaga, Abraham K Isaak, Sana Zainab, Mohammedsefa Arusi Dari, Martha Mekonen Gdey, Areeba Khan

**Affiliations:** 1 Medicine, National Autonomous University of Honduras, San Pedro Sula, HND; 2 Medicine, American College of Physicians, Philadelphia, USA; 3 Interventional Cardiology, Himalayan Institute of Medical Sciences, Dehradun, IND; 4 Medicine, National Autonomous University of Honduras, Tegucigalpa, HND; 5 Telemetry, Sharp Memorial Hospital, San Diego, USA; 6 Internal Medicine, Orotta School of Medicine and Dentistry, Asmara, ERI; 7 Medicine, Khalifa University, Abu Dhabi, ARE; 8 Head and Neck Surgery, Addis Ababa University, Addis Ababa, ETH; 9 General Practice, Mekelle University, Mek'ele, ETH; 10 Critical Care Medicine, United Medical and Dental College, Karachi, PAK

**Keywords:** systematic review and meta-analysis, efficacy, myocardial infarction, angiotensin-converting enzyme inhibitors, angiotensin ii receptor blockers

## Abstract

Patients with acute myocardial infarction (AMI) are usually treated with angiotensin-converting enzyme inhibitors (ACEi) or angiotensin II receptor blockers (ARB). The aim of this meta-analysis is to compare outcomes between ACEi and ARB in patients with myocardial infarction (MI). This meta-analysis was conducted in accordance with the Preferred Reporting Items for Systematic Reviews and Meta-Analyses (PRISMA) 2020 guidelines. Three major online databases, including PubMed, EMBASE, and the Cochrane Library, were thoroughly searched to find studies comparing ACEi and ARB in patients with MI from January 1, 2000, onwards, without language or publication restrictions. Outcomes assessed in this meta-analysis included major adverse cardiovascular events (MACE), all-cause mortality, cardiovascular mortality, stroke, and hospitalization due to heart failure. A total of 16 studies were included in this meta-analysis. Pooled estimates showed no significant differences between the two groups in terms of MACE (risk ratio (RR): 1.03, 95% confidence interval (CI): 0.88-1.20), all-cause mortality (RR: 1.03, 95% CI: 0.88-1.20), cardiovascular mortality (RR: 1.00, 95% CI: 0.89-1.12), stroke (RR: 1.03, 95% CI: 0.80-1.32), and hospitalization due to heart failure (RR: 0.99, 95% CI: 0.90-1.09). These results suggest that ACEi and ARB have similar impacts on clinical outcomes across a broad spectrum of MI patients, reinforcing their roles in post-MI treatment.

## Introduction and background

Individuals who experience acute myocardial infarction (AMI) accompanied by significant myocardial damage often display clinical signs of heart failure or left ventricular dysfunction, putting them at a heightened risk of health complications and mortality [1]. To address these concerns, both angiotensin-converting enzyme inhibitors (ACEi) and angiotensin II receptor blockers (ARBs) have been recommended as preventive treatments for AMI patients in the guidelines provided by the European Society of Cardiology and the American College of Cardiology/American Heart Association (ACC/AHA) [2-3].

ACEi were the first renin-angiotensin-aldosterone system (RAS) inhibitors to be clinically approved, and substantial evidence from the 1990s and early 2000s established their efficacy in reducing morbidity and mortality related to cardiovascular diseases [4-5]. In 1995, ARBs became available for clinical use, and their effectiveness for similar indications has been well-documented [6]. Notably, while the risk of hyperkalemia and renal dysfunction associated with treatment is comparable between these two drug classes, ARBs are less likely to induce cough, even in patients who experienced this side effect while taking ACEi [7].

ACEi have traditionally been recommended as the standard treatment for individuals with myocardial infarction (MI), especially in cases involving left ventricular systolic dysfunction, diabetes, or chronic kidney disease [8]. However, it is important to note that ACEi do not entirely block the production of angiotensin II. This limitation has prompted the consideration that direct receptor blockade might offer a more effective approach. It has been demonstrated that angiotensin II can also be generated through a pathway that is independent of ACE, involving chymase, and this pathway is significant in humans [9]. Incomplete inhibition of angiotensin II production has been reported with low-dose and long-term use of ACEi [10]. Hence, the use of ARBs may present a more comprehensive strategy for completely thwarting the harmful effects of angiotensin II, specifically at the type 1 receptor level. ARBs are known to have additional favorable effects on cardiovascular function and structure by stimulating the type 2 receptor [11]. Furthermore, in experimental models of post-MI heart failure, ARBs have demonstrated the ability to reduce MCP-1 expression and macrophage infiltration in the border zone, resulting in less myocardial fibrosis [12].

Previous investigations have generated conflicting findings when comparing the efficacy and safety of ACEi and ARB in patients with a history of MI. Therefore, conducting a comprehensive and rigorous meta-analysis is essential to thoroughly assess and contrast the performance of these two drug classes. This meta-analysis has been undertaken with the objective of comparing the outcomes between ACEi and ARB in patients who have experienced MI.

## Review

Methodology

This meta-analysis was conducted in accordance with the Preferred Reporting Items for Systematic Reviews and Meta-Analyses (PRISMA) 2020 guidelines.

Search Strategy

Three major online databases, including PubMed, EMBASE, and the Cochrane Library, were thoroughly searched to find studies comparing ACEi and ARB in patients with MI from January 1, 2000, onwards, without language or publication restrictions. Keywords used to search for relevant articles included "Angiotensin-converting enzyme inhibitor," "angiotensin receptor blockers," "myocardial infarction," and their synonyms. To further optimize the search, medical subject headings (MeSH) terms were used along with Boolean algebra operators. Additionally, reference lists of all included articles were manually screened to find additional studies relevant to the study topic.

Study Selection

Two authors independently screened all studies using titles and abstracts, followed by a full-text review according to the selection criteria. Any disagreements in the study selection process were resolved through consensus. Only original studies (randomized controlled trials (RCTs) and observational studies) were included, with reviews, letters, conference abstracts, and study protocols excluded from this meta-analysis. We included studies comparing ACEi and ARB in patients with MI. We excluded studies comparing either of these two drugs with a placebo or any other drug. Studies assessing the effectiveness of these drugs in patients other than those with MI were also excluded. We also excluded studies that did not report outcomes assessed in the present meta-analysis.

Data Extraction and Outcomes

Data from the included studies were extracted using a pre-designed Excel spreadsheet. Two authors extracted relevant data from all included studies. The extracted data included author names, year of publication, region, study design, sample size, follow-up duration, characteristics of participants, and outcomes. Outcomes assessed in this meta-analysis included major adverse cardiovascular events (MACE), all-cause mortality, cardiovascular mortality, stroke, and hospitalization due to heart failure.

Statistical Analysis

Data analysis was performed using RevMan version 5.4.1 (The Cochrane Collaboration, London, United Kingdom). To compare outcomes between ARB and ACEi, we reported risk ratios (RR) with 95% confidence intervals (CI). A p-value <0.05 was considered statistically significant. Heterogeneity among the study results was assessed using I-square, with I-square >50% considered significant for heterogeneity. In such cases, a random-effect model was used. Otherwise, a fixed-effect model was used to calculate effect estimates. Subgroup analysis was performed based on follow-up duration and study design.

Results

**Figure 1:**
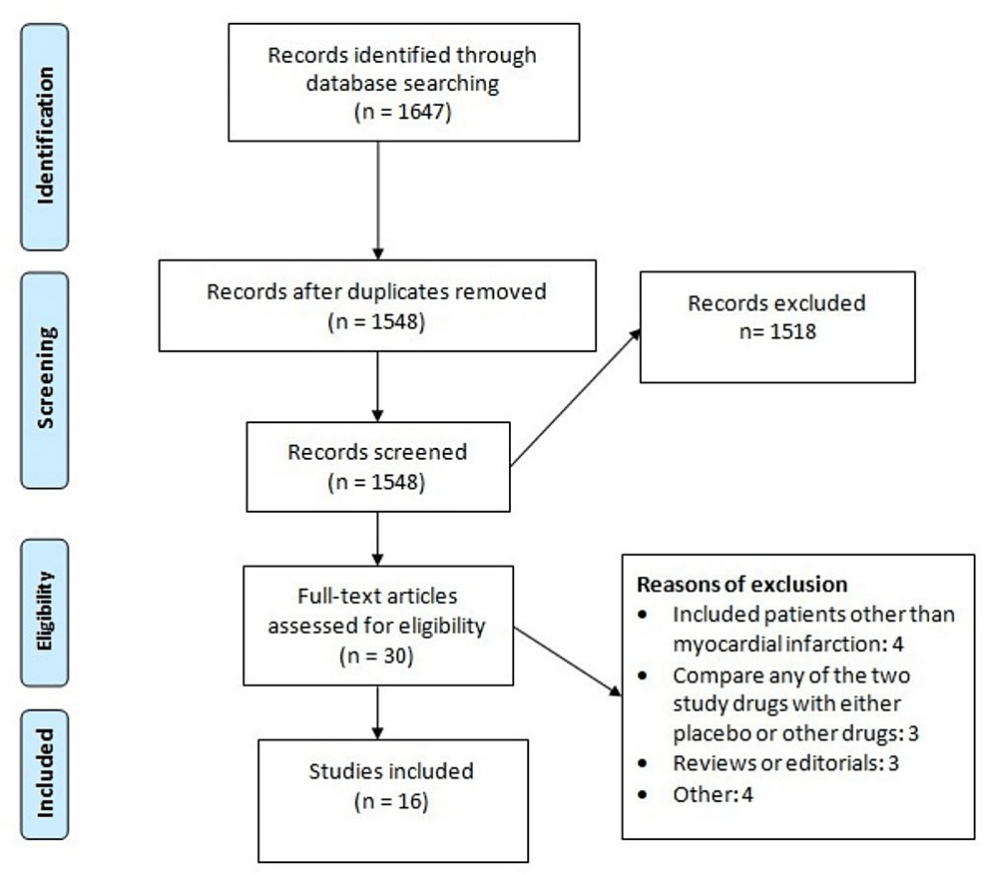
PRISMA flowchart of the study selection

Figure 1 shows the process of study selection. A total of 1,647 records were initially identified through the initial search strategy. After removing duplicates, titles, and abstracts, 1,548 studies were screened, followed by a detailed assessment of the full text of the articles using pre-defined inclusion and exclusion criteria. In total, 16 studies were included in this meta-analysis. Table 1 shows the characteristics of the included studies [1,13-27]. Three studies were RCTs [1,24,27]. The follow-up of included studies ranged from six months to 60 months.

**Table 1:** Characteristics of the included studies RCT: Randomized controlled trial; ARB: angiotensin II receptor blockers; ACEi: Angiotensin-converting enzyme inhibitors; NR: Not reported

Author Name	Year	Study Design	Region	Groups	Sample Size	Follow-up	Age (Years)	Males (n)	Diabetes (n)	Hypertension (n)
Byun et al. [13]	2018	Observational	Korea	ARB	1103	24 Months	66	697	1,103	807
				ACEi	1103		67	686	1,103	793
Chen et al. [14]	2023	Observational	Taiwan	ARB	14993	18 Months	81.5	12,219	5,173	10,540
				ACEi	14993		81.5	12,219	5,248	10,420
Choi et al. [15]	2019	Observational	Korea	ARB	2822	12 Months	NR	NR	NR	NR
				ACEi	2811					
Dickstein et al. [1]	2002	RCT	Multinational	ARB	2744	32.4 Months	67.6	1,969	488	983
				ACEi	2733		67.2	1,933	452	987
Hara et al. [16]	2014	Observational	Japan	ARB	2158	60 Months	67	1,597	734	1,517
				ACEi	4425		65	3,447	1,443	2,624
Her et al. [17]	2020	Observational	Korea	ARB	1359	36 Months	60.8	1,130	261	0
				ACEi	2634		59.7	2,227	454	0
Hyun et al. [18]	2023	Observational	Korea	ARB	534	36 Months	64.2	388	152	283
				ACEi	534		64.2	390	154	279
Kim et al. [19]	2021	Observational	Korea	ARB	2197	12 Months	63.5	1,637	627	1,230
				ACEi	2945		61.9	2,135	660	1,388
Ko et al. [20]	2019	Observational	United States	ARB	17227	36 Months	77.1	10,250	7,856	15,573
				ACEi	42126		77	25,107	19,167	38,082
Lee et al. [21]	2016	Observational	Korea	ARB	715	12 Months	65.8	488	206	366
				ACEi	2767		65.7	1,951	801	1,481
Lee et al. [22]	2023	Obervational	Korea	ARB	1967	24 Months	67.1	1,290	768	1,967
				ACEi	1967		66.9	1,305	747	1,967
Lim et al. [23]	2019	Observational	Korea	ARB	836	43.8 Months	61.9	585	277	464
				ACEi	824		58.3	682	233	336
Pfeffer et al. [24]	2003	RCT	Multi national	ARB	4909	24.7 Months	65	3,365	1,146	2,700
				ACEi	4909		64.9	3,373	1,134	2,732
She et al. [25]	2021	Observational	China	ARB	291	24 Months	62.13	226	NR	NR
				ACEi	291		61.98	228		
Song et al. [26]	2015	Observational	Korea	ARB	1171	12 Months	66.6	817	648	338
				ACEi	2752		66.2	1,953	1,477	789
Suzuki et al. [27]	2009	RCT	Japan	ARB	120	6 Months	63	101	41	73
				ACEi	121		62.9	99	41	65

Major Adverse Cardiovascular Events (MACE)

**Figure 2:**
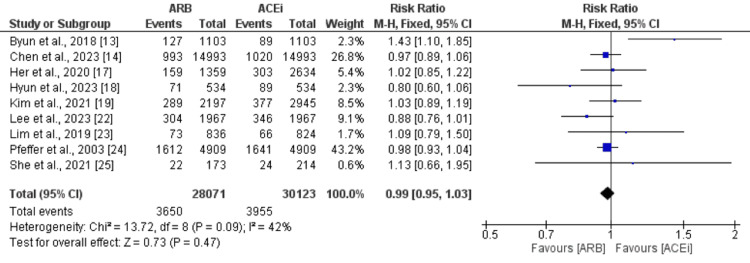
Comparison of MACE between ARB and ACEi ARB: angiotensin II receptor blockers; ACEi: Angiotensin-converting enzyme inhibitors Sources: References [13-14,17-19,22-25]

Nine studies were included in the pooled analysis of the comparison of MACE between patients who received ARB and ACEi. The number of MACE events was slightly higher in patients receiving ACEi (13.12%) compared to patients receiving ARB (13.07%), but the difference was statistically insignificant (RR: 0.99, 95% CI: 0.95-1.03), as shown in Figure 2. No significant heterogeneity was reported among the study results (I-square: 42%).

All-Cause Mortality

**Figure 3:**
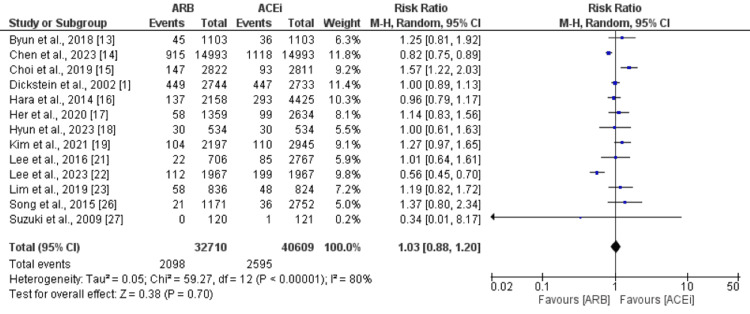
Comparison of all-cause mortality between ARB and ACEi ARB: angiotensin II receptor blockers; ACEi: Angiotensin-converting enzyme inhibitors Sources: References [1,12,14-19,21-23,26-27]

Thirteen studies were included in the pooled analysis comparing the risk of all-cause mortality among patients who received ARB and ACEi. Pooled analysis showed that all-cause mortality was not significantly different between ARB and ACEi groups (RR: 1.03, 95% CI: 0.88-1.20), as shown in Figure 3. High heterogeneity was reported among the study results (I-square: 80%).

Cardiovascular Mortality

**Figure 4:**
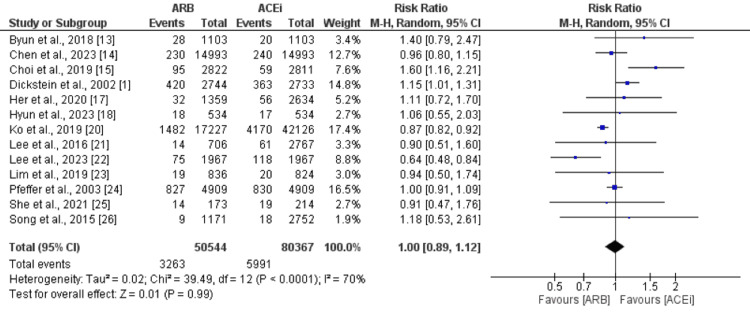
Comparison of cardiovascular mortality between ARB and ACEi ARB: angiotensin II receptor blockers; ACEi: Angiotensin-converting enzyme inhibitors Sources: References [1,13-15,17-18,20-26]

Thirteen studies were included in the pooled analysis of the comparison of cardiovascular death between ARB and ACEi groups. As shown in Figure 4, no significant difference was observed between the two groups in terms of cardiovascular death (RR: 1.00, 95% CI: 0.89-1.12). High heterogeneity was reported among the study results (I-square: 70%).

Stroke

**Figure 5:**
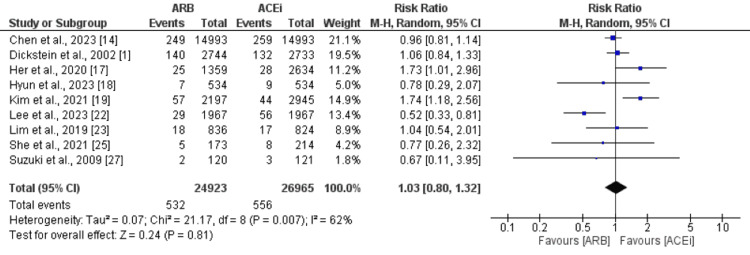
Comparison of stroke between ARB and ACEi ARB: angiotensin II receptor blockers; ACEi: Angiotensin-converting enzyme inhibitors Sources: References [1,14,17-19,22-23,25,27]

Nine studies were included in the pooled analysis compared the risk of stroke between ARB and ACEi groups. As shown in Figure 5, the risk of stroke was not significantly different between patients who received ARB and ACEi (RR: 1.03, 95% CI: 0.80-1.32). High heterogeneity was reported among the study results (I-square: 62%).

HF Hospitalization

**Figure 6:**
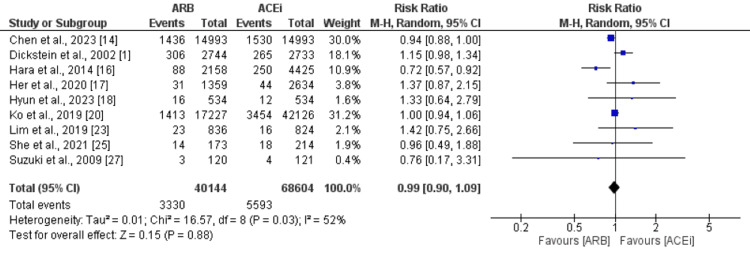
Comparison of HF hospitalization between ARB and ACEi ARB: angiotensin II receptor blockers; ACEi: Angiotensin-converting enzyme inhibitors Sources: References [1,14,16-18,20,23,25,27]

Nine studies were included in the pooled analysis comparing the risk of HF hospitalization between ARB and ACEi groups. No significant difference was reported between the two groups in relation to the risk of HF hospitalization (RR: 0.99, 95% CI: 0.90-1.09), as shown in Figure 6. Significant heterogeneity was reported among the study results (I-square: 52%).

Subgroup Analysis

The analysis of MACE demonstrated no significant differences in risk between ACE inhibitors and ARBs, whether the follow-up duration was less than or greater than one year, or when considering the study design (RCT or observational). For all-cause mortality, the risk was elevated in the shorter-term follow-up (<=1 year) when compared to the longer-term (>1 year), but the difference was not statistically significant. Interestingly, in observational studies, all-cause mortality showed a higher risk with ARBs. Cardiovascular mortality outcomes displayed no significant differences across the subgroups based on follow-up duration or study design (Table 2).

**Table 2:** Subgroup analysis MACE: Major adverse cardiovascular events; RCT: Randomized controlled trial; RR: risk ratio; CI: confidence interval

Outcomes	Groups	RR (95% CI)
MACE	<=1 Year	0.95 (0.86-1.05)
	> 1 Year	0.98 (0.94-1.02)
All-cause Mortality	<=1 Year	1.35 (1.15-1.59)
	> 1 Year	0.90 (0.74-1.08)
Cardiovascular Mortality	<=1 Year	1.28 (0.88-1.87)
	> 1 Year	0.95 (0.84-1.07)
MACE	RCT	0.98 (0.93-1.04)
	Observational	0.97 (0.91-1.02)
All-cause Mortality	RCT	1.00 (0.89-1.13)
	Observational	1.04 (0.83-1.32)
Cardiovascular Mortality	RCT	1.06 (0.92-1.22)
	Observational	0.96 (0.82-1.14)

Discussion

This meta-analysis of various studies comparing ACEi and ARB in patients with MI revealed no significant differences between the two groups in terms of MACE, all-cause mortality, cardiac mortality, stroke, and hospitalization due to heart failure. These findings indicate that ACEi and ARB have comparable impacts on clinical outcomes across a broad spectrum of MI patients.

Given the heterogeneity of MI patients with varying cardiac event risks, clarifying the roles of ARB versus ACEi in this diverse patient group is essential. Our results confirm and extend previous trials, a meta-analysis, and recent ARB and ACEi guidelines [2,28-29], suggesting that ARB may offer a valuable option for reducing mortality and morbidity in a wide range of MI patients.

ARB are recommended for patients intolerant to ACEi. For example, the ARB valsartan demonstrated approximate equivalence to the ACEi captopril in the VALIANT trial, and the ARB telmisartan was comparable to the ACEi ramipril in patients with vascular disease or high-risk diabetes in the ONTARGET trial [28-29]. In the context of acute MI, both ACEi and ARB have proven their survival benefits in various randomized trials, significantly reducing fatal and non-fatal major cardiovascular events [29-30]. ARB use has also shown substantial therapeutic benefits equivalent to those of ACEi [28].

In this meta-analysis, we conducted subgroup analyses based on follow-up duration (<=1 year and >1 year) and observed no significant differences between ACEi and ARB in terms of MACE events, all-cause mortality, and cardiovascular mortality. The choice between ACEi and ARB often depends on the specific patient population and indications, as these drugs are prescribed for various conditions such as hypertension, heart failure, and diabetic nephropathy. This choice can be influenced by individual patient characteristics and comorbidities.

Several studies have compared the occurrence of MACE events in different patient groups. For instance, the study conducted by Lee et al. [22] reported no significant difference in the effects of ACEi and ARB in patients with or without diabetes, with reduced or normal left ventricular ejection fraction (LVEF), and patients with or without coronary artery disease (CAD). However, these studies are observational and may be subject to bias. Therefore, future RCTs are needed to compare the effects of ACEi and ARB in MI patients with different comorbidities to determine which drug is most beneficial in terms of preventing cardiovascular events.

While ACEi are effective in reducing adverse cardiovascular events, ARB were developed to block angiotensin II through pathways independent of ACE. Unlike ACEi, ARB do not hinder the breakdown of bradykinin, which results in increased tolerance in patients, reducing the risk of cough or angioedema. However, for individuals experiencing acute MI, current guidelines suggest considering ARBs as an alternative for those who cannot tolerate ACEi. This limited recommendation for ARBs in AMI patients is based on several factors. ARBs are not expected to offer a superior advantage over the well-established benefits of ACEi; rather, they are considered to provide a non-inferior beneficial effect [7,24]. Furthermore, prior research has yielded inconclusive findings; for example, losartan failed to meet the non-inferiority standard [1]. ARBs are associated with higher costs, a tendency to induce greater hypotension, and have been linked to renal dysfunction in randomized trials [31-32]. Therefore, ACEi should be the preferred class of drugs among RAS inhibitors, with ARB primarily serving as an alternative option for patients with AMI who cannot tolerate ACEi.

Data regarding the beneficial impacts of the alternative use of ARBs in low-risk myocardial patients are insufficient. While ACEi are recommended for all AMI patients, the strength of this recommendation varies depending on the patient's risk profile. The class of recommendation for low-risk patients, such as those with preserved left ventricular systolic function and no hypertension or diabetes, is less robust compared to that for high-risk patients. Furthermore, there is a lack of clinical data on the use of ARBs in the treatment of AMI, especially in low-risk patient populations or specific subgroups. Recent studies based on registry data have produced conflicting results regarding the clinical impact of ARBs in AMI patients [14,16,33]. However, the present study supports the use of ARBs in patients with MI. Nevertheless, it is important to note that the findings of this meta-analysis are based on observational studies. Further large-scale nationwide data or randomized trials are needed.

The current meta-analysis has certain limitations. Firstly, most of the included studies were observational in nature. Observational studies can be more susceptible to various types of bias, such as selection bias, confounding, and measurement bias. Therefore, in the future, more trials are required to gain a better understanding of the drugs that provide maximum benefits to patients with MI. Secondly, most of the studies did not report specific doses and compliance, as well as the incidence of discontinuation. These are important aspects to consider when reporting the efficacy of the drugs.

## Conclusions

In conclusion, this meta-analysis, which involved a comprehensive evaluation of numerous studies, aimed to compare ACEi and ARB in patients with MI. The analysis, which included a substantial number of articles after thorough screening, found no significant differences between ACEi and ARB with regard to MACE, all-cause mortality, cardiac mortality, stroke, and hospitalization due to heart failure. These results suggest that ACEi and ARB have similar impacts on clinical outcomes across a broad spectrum of MI patients, reinforcing their roles in post-MI treatment. While the inclusion of diverse patient groups within the AMI spectrum underscores the importance of assessing ARBs versus ACEIs, it is essential to recognize the context-specific nature of these drug choices, influenced by patient characteristics and comorbidities.
